# Adaptive Causal Thinking About Mobility Challenges: Implications for Quality of Life

**DOI:** 10.1093/geroni/igaa057.951

**Published:** 2020-12-16

**Authors:** Judith Chipperfield, Jeremy Hamm, Patricia Parker, Maria Krylova, Loring Chuchmach, Raymond Perry, Cheliza Krause, Christiane Hoppmann

**Affiliations:** 1 University of Manitoba, Winnipeg, Manitoba, Canada; 2 North Dakota State University, Fargo, North Dakota, United States; 3 University of British Columbia, Vancouver, British Columbia, Canada

## Abstract

Weiner’s attribution theory posits that it is adaptive to ascribe challenges to controllable causes (e.g., insufficient effort, bad strategies) and maladaptive to ascribe them to uncontrollable causes (e.g., old age). This is supported by our prior research that showed a heightened risk of mortality when mobility challenges were attributed to old age. The present pilot study randomly assigned older adults (N=36) in a day hospital to either an attributional retraining (AR) intervention group that viewed a video intended to shift causal thinking regarding mobility challenges (uncontrollable→controllable causes), or to a comparison group (No-AR). Participants completed a Time1 survey, the AR intervention (one week later), and a Time2 follow-up survey two weeks later. A manipulation-check revealed that AR was effective in shifting causal thinking away from maladaptive causes; a decline in the endorsement of the old age attribution was observed in the AR group (Ms=2.61 vs. 2.06; p=.02), but not in the No-AR group (Ms=2.45 vs. 2.35, p=.30). The AR and No-AR groups were equivalent at Time 1 on two quality-of-life outcomes: helplessness and perceived control (PC) over health. However, helplessness declined (Time1-Time2) in the AR group (Ms=1.13 vs. 0.73, p=.03), whereas it was relatively stable in the No-AR group (Ms=1.42 vs. 1.26, p>.20). Moreover, PC increased marginally in the AR group (Ms=6.50 vs. 6.69; p=.06), but declined in the No-AR group (Ms=6.20 vs 5.45, p=.05). Together, these findings suggest that attributions can be shifted away from uncontrollable causes and that this shift can have a protective effect that benefits quality-of-life.

